# Crystal Structure of Hyp-1, a *Hypericum perforatum* PR-10 Protein, in Complex with Melatonin

**DOI:** 10.3389/fpls.2016.00668

**Published:** 2016-05-18

**Authors:** Joanna Sliwiak, Zbigniew Dauter, Mariusz Jaskolski

**Affiliations:** ^1^Center for Biocrystallographic Research, Institute of Bioorganic Chemistry, Polish Academy of SciencesPoznan, Poland; ^2^Synchrotron Radiation Research Section, National Cancer Institute, Argonne National Laboratory, ArgonneIL, USA; ^3^Department of Crystallography, Faculty of Chemistry, Adam Mickiewicz University in PoznańPoznań, Poland

**Keywords:** pathogenesis-related protein, PR-10, phytohormone, ligand binding, cytokinin

## Abstract

Hyp-1, a PR-10-fold protein from *Hypericum perforatum*, was crystallized in complex with melatonin (MEL). The structure confirms the conserved protein fold and the presence of three unusual ligand binding sites, two of which are internal chambers (1,2), while the third one (3) is formed as an invagination of the protein surface. The MEL ligand in site 1 is well defined while that in site 3 seems to be rotating between the side chains of Lys33 and Tyr150 that act as a molecular vise. The patch of electron density in site 2 does not allow unambiguous modeling of a melatonin molecule but suggests a possible presence of its degradation product. This pattern of ligand occupation is reproducible in repeated crystallization/structure determination experiments. Although the binding of melatonin by Hyp-1 does not appear to be very strong (for example, MEL cannot displace the artificial fluorescence probe ANS), it is strong enough to suggest a physiological role of this interaction. For example, *trans*-zeatin, which is a common ligand of PR-10 proteins, does not overcompete melatonin for binding to Hyp-1 as it does not affect the crystallization process of the Hyp-1/MEL complex, and among a number of potential natural mediators tested, melatonin was the only one to form a crystalline complex with Hyp-1 with the use of standard crystallization screens. Hyp-1 is the second protein in the Protein Data Bank for which melatonin binding has been demonstrated crystallographically, the first one being human quinone reductase.

## Introduction

Hyp-1, the protein product (comprised of 159 residues) of the *hyp-1* gene in *Hypericum perforatum* has a picturesque history. It was first described as the enzyme catalyzing the biosynthesis of the pharmacological ingredient of this plant, the dianthrone hypericin, from two molecules of emodin (Bais et al., 2003). That study, however, could never be replicated and instead the crystal structure of the Hyp-1 protein revealed the canonical PR-10-fold (Michalska et al., 2010) strongly suggesting classification in class 10 of the superfamily of plant Pathogenesis-Related (PR-10) proteins. The latter hypothesis was corroborated by genetic data, which showed that *hyp-1* has gene structure analogous to typical *pr-10* genes (Kosuth et al., 2013), but it has to be underlined that Hyp-1 has not been demonstrated so far to be involved in stress response of *H. perforatum*. With regard to the localization in the plant, it was shown that Hyp-1 mRNA expression occurs in all organs of *Hypericum* seedlings with the highest levels in roots (Kosuth et al., 2007), whereas immunofluorescence assays of plantlets revealed wide distribution of the Hyp-1 protein in different tissues, including roots, stem, and leaves (Qian et al., 2012).

**FIGURE 1:**
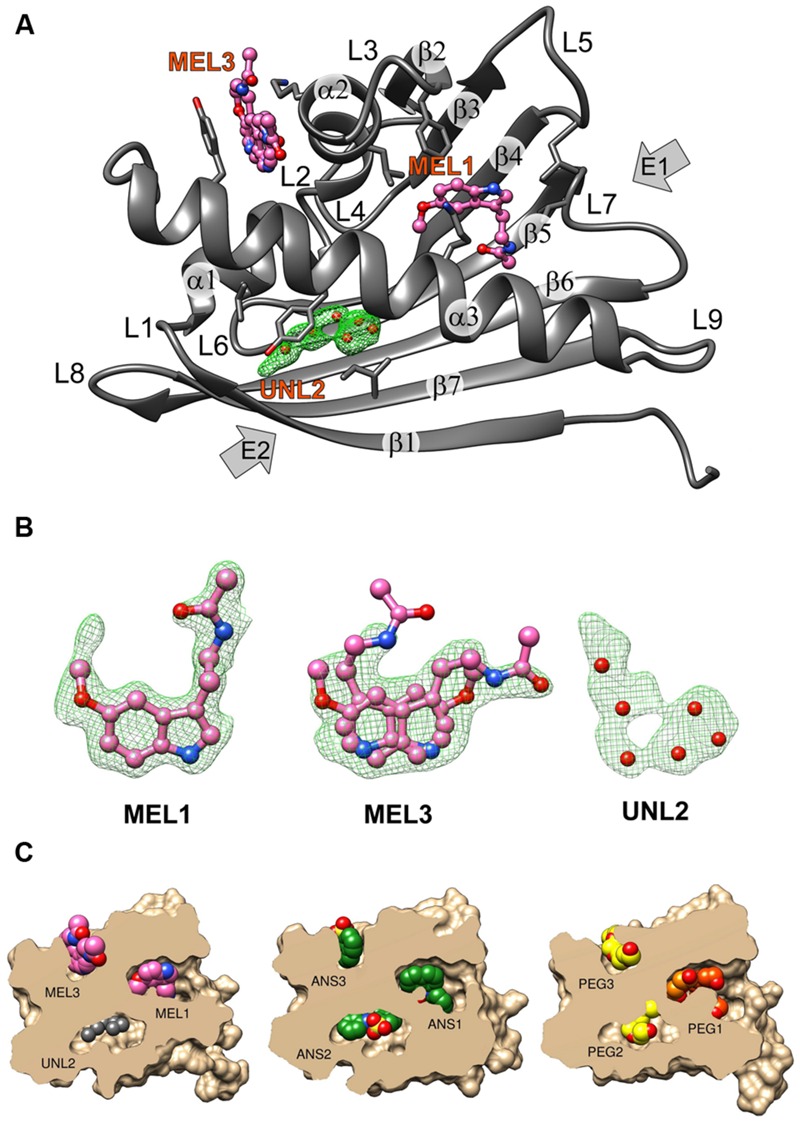
**(A)** The crystal structure of the Hyp-1/MEL complex with annotation of the canonical structural elements of the PR-10-fold. Residues that are in contact with the ligand molecules at <3.8 Å are shown as sticks. MEL molecules 1 and 3 are shown in ball-and-stick representation whereas the unknown ligand at site 2 is represented by its OMIT *F_o_–F_c_* electron density contoured at 2.5σ and marked with six dummy water molecules (red spheres). **(B)** *F_o_–F_c_* OMIT maps contoured at 2.5σ corresponding to MEL1 at site 1, MEL3 at site 3 and UNL at site 2. **(C)** Structures of the Hyp-1 complexes with MEL (this work), ANS (4N3E, chain K), and PEG (3IE5, chain B) shown in cutaway surface representation. The ligand molecules are shown as van der Waals models. “PEG” denotes various fragments (oligomers) of polyethylene glycol, a buffer component that was serendipitously bound by the Hyp-1 protein in the experiments conducted by Michalska et al. (2010).

The characteristic PR-10-fold (Fernandes et al., 2013), also known as the Betv1 fold according to the first protein from this class, a birch (*Betula verucosa*) pollen allergen to have its crystal structure determined (Gajhede et al., 1996), consists of a large seven-stranded antiparallel β-sheet forming a baseball-glove grip over a long C-terminal helix α3, which is the most variable element of the PR-10 structure (Biesiadka et al., 2002; Pasternak et al., 2006). The consecutive β-strands are connected by loops, except for strands β1 (first) and β2 (last) at the edges of the β-sheet, which are connected by a V-shaped fork of two α-helices (α1 and α2) that provides a support for the C-terminal end of helix α3. At its N-terminal end, helix α3 is connected to the protein scaffold by loop L9. A conspicuous feature of the PR-10-fold is a large hydrophobic cavity formed between the main structural elements, i.e., the β-sheet and helix α3, with the participation of other secondary structures, such as the odd-numbered loops (L3, L5, L7, L9), which are the fingertips of the gripping hand (**Figure 1A**). The cavity has two entrances connecting it to the outer environment: E1 surrounded by the odd numbered loops (L3, L5, L7) and helix α3, and entrance E2 located between helix α3 and strand β1. Despite the hollow core, the PR-10 proteins are robust, resistant to proteases and have mechanical stability that even surpasses that of average globular proteins (Chwastyk et al., 2014). The properties, size and shape of the internal cavity are mostly modulated by the character of the α3 helix.

The presence of such an intriguing cavity has led to the hypothesis that PR-10 proteins might have evolved in plants to bind/store/transport important small-molecule mediators, such as plant hormones (Fernandes et al., 2013). Along these lines, a number of PR-10 (or at least PR-10-fold) proteins have been characterized structurally in complex with phytohormones (or their analogs), such as cytokinins (Pasternak et al., 2006; Fernandes et al., 2008, 2009; Kofler et al., 2012; Ruszkowski et al., 2013; Sliwiak et al., 2016), gibberellin (Ruszkowski et al., 2014), brassinosteroids (Markovic-Housley et al., 2003), or abscisic acid (Sheard and Zheng, 2009). Moreover, other plant metabolites, such as flavonoids (Mogensen et al., 2002; Kofler et al., 2012; Casañal et al., 2013) or their glycosylated forms (Seutter von Loetzen et al., 2014, 2015), are also bound by PR-10 proteins.

**FIGURE 2:**
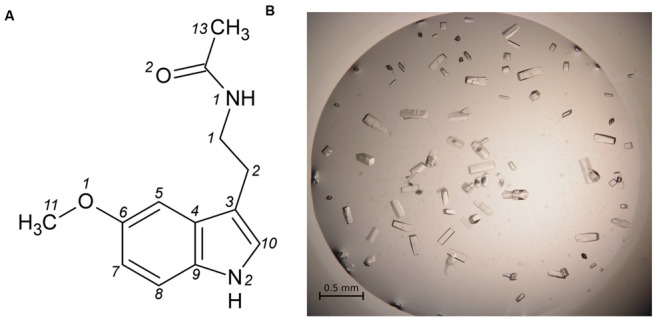
**(A)** Melatonin with atom numbering according to Quarles et al. (1974). **(B)** Single crystals of the Hyp-1/MEL complex grown in 1 M citrate and 20% glycerol.

On the list of recognized plant hormones, melatonin (*N*-acetyl-5-methoxytryptamine, MEL, **Figure 2A**) is a relatively new addition. Apart from the discovery of the presence of this conservative molecule in plants (Dubbels et al., 1995), relatively little has been learned about phytomelatonin function over the last decade. Melatonin appears to regulate plant growth in an auxin-like manner, regulate the response to photoperiod and increase tolerance to abiotic stress. It is also one of the most efficient antioxidants (Arnao and Hernández-Ruiz, 2015). *H. perforatum*, alongside *Tanacetum parthenium* or the Chinese herb *Scuttelaria biacalensis*, appears to contain very high MEL concentrations (Murch et al., 1997) that reach 2 μg/g of dried weight in leaves and are above 4 μg/g in flowers. In the case of *H. perforatum*, this could be responsible for the medicinal effects of St John’s wort preparations. Apart from tissue content determinations and studies of the tryptophan-dependent biosynthetic pathway (Murch et al., 2000), studies also focused on the role of melatonin in *H. perforatum*, demonstrating that increased light intensity elevates melatonin synthesis, thus confirming its free radical scavenging function (Murch et al., 2000). It was shown that MEL is able to induce rhizogenesis (Murch et al., 2001), an observation that has been recently confirmed in other plant species (Arnao and Hernández-Ruiz, 2015).

Biophysical and kinetic studies of PR-10/phytohormone complexes are often difficult because of problems with ligand solubility, low heat effect upon binding (in calorimetry) and/or unsuitable spectroscopic properties. A frequently used assay in such studies is ADA (ANS Displacement Assay), in which the fluorescent dye 8-anilinonaphthalene-1 sulfonate (ANS) is displaced by the ligand of interest (Mogensen et al., 2002). Our investigations of ligand-binding properties of the Hyp-1 protein started in fact with the crystallization of a Hyp-1/ANS complex, which turned out to have a complex modulated crystal structure with as many as 28 copies of the protein in the asymmetric unit (Sliwiak et al., 2014, 2015). At the same time, that structure revealed an unprecedented among PR-10-fold proteins ligand binding mode, with two ANS molecules (at sites 1, 2) bound in two tight internal chambers (instead of one large cavity) and another one (3) docked in a deep invagination of the protein surface.

In this work we present high resolution crystal structure of Hyp-1 in complex with melatonin, demonstrating that this physiological ligand utilizes the same internal docking sites as ANS. The Hyp-1/MEL structure is the first example reported in the Protein Data Bank (PDB) of melatonin bound to a plant protein, and the second case with any protein, the first one being human quinone reductase (Calamini et al., 2008). It is also important to stress that among many different phytohormones and hypothetical biologically relevant substrate/product molecules (e.g., emodin, hypericin) tested, melatonin was the only ligand that formed crystalline complex with the Hyp-1 protein.

## Materials and Methods

### Protein Preparation, Complex Formation, and Crystallization

Hyp-1 was produced as described before (Sliwiak et al., 2015). Prior to crystallization, the protein solution was concentrated to 15 mg/ml and pre-incubated at 292 K for 1 h with 10-fold molar excess of melatonin (Sigma–Aldrich) added from a 0.1 M stock solution in methanol, or with MEL powder. Screening for Crystal Screen, PEG/Ion I and II (Hampton Research) crystallization conditions was performed by the sitting drop vapor diffusion method against 120 μL well solution with the use of a Mosquito Crystallization Robot. The crystallization drops were mixed from 0.2 μL protein/ligand solution and 0.2 μL well solution. Small crystals, which appeared the same day in 1.6 M tribasic sodium citrate, pH 6.5, were used for seeding in a gradient of PEG 400 or glycerol and tribasic sodium citrate in hanging drops. Large, prismatic crystals of dimensions 0.08 mm × 0.08 mm × 0.2 mm (**Figure 2B**) grew in 1 M citrate and 20% glycerol.

### Competitive Crystallization Assays

Hyp-1 protein was pre-incubated for 1 h with an equimolar solution of MEL (from 0.1 M methanol stock) and *trans*-zeatin (from 0.1 M stock in DMSO), as well as with a solution mixture of MEL and ANS (from 0.1 M stock in DMSO), mixed at the following MEL:ANS ratios: 1:1, 2:1, and 3:1. The Hyp-1:MEL molar ratio was 1:10 in all conditions. Crystallization was performed in all these cases using the final growth conditions established for the crystals of the Hyp-1/MEL complex. In these competition assays, crystals were obtained only in the presence of *trans*-zeatin but they had the prismatic morphology of the Hyp-1/MEL crystals.

### Data Collection, Structure Solution, and Refinement

X-Ray diffraction data extending to 1.30 Å resolution were collected at the SER-CAT 22ID beamline of the Advanced Photon Source (APS/ANL) and were processed with HKL-2000 (Otwinowski and Minor, 1997). The data were merged in space group *C*222_1_ with *R*_merge_ of 5.7% (**Table 1**). For molecular replacement in Phaser (McCoy et al., 2007), the PDB model 3IE5 (Michalska et al., 2010) was used. Manual rebuilding was carried out in Coot (Emsley et al., 2010) and anisotropic maximum-likelihood refinement was carried out in phenix.refine (Afonine et al., 2012). Stereochemical restraints for the melatonin molecule were generated from the coordinates found in the CSD deposit MELATN01 of melatonin crystal structure (Quarles et al., 1974). X-Ray diffraction data collected for identical crystals, obtained upon co-crystallization with melatonin added in pulverized form or in the presence of equimolar concentration of *trans*-zeatin, extended to 1.34 Å and 1.40 Å resolution, respectively, and were also merged in the *C*222_1_ space group with *R*_merge_ of 7.8 and 9.6%, respectively.

**Table 1:** Data collection and refinement statistics.

**Data collection**	
Space group	*C*222_1_
Unitcell parameters *a, b, c* (Å)	60.86, 89.64, 76.41
Beamline	SER-CAT 22ID (APS)
Wavelength (Å)	1.0000
Data collection temperature (K)	100
Resolution (Å)	30.0–1.30 (1.32–1.30)^a^
*R*_merge_ (%)	5.7 (51.4)
<*I/*σ*I* >	28.9 (2.5)
CC_1/2_/CC^∗^ (%)^b^	(86.3)/(96.2)
Completeness (%)	99.9 (99.6)
Redundancy	4.9 (3.9)
**Refinement**	
Resolution (Å)	25.31–1.30
Reflections work/test	49014/2630
*R*_work_*/R*_free_ (%)	12.8/15.3
Protein/ligand/solvent/water/metal atoms	1405/51(MEL), 12(GOL), 6(UNL)/204/3
<*B*> protein/ligand/water/metal (Å^2^)	22.8/45.3(MEL), 58.8(GOL), 59.8(UNL)/45.2/37.8
R.M.S.D. from ideal geometry	
Bond lengths (Å)/bond angles (^o^)	0.017/1.6
Ramachandran statistics (%)^c^	
Favored/outliers	98.3/0
PDB code	5I8F

*^a^Values in parentheses correspond to the last resolution shell. ^b^Correlation coefficients, as defined by Karplus and Diederichs (2012), given for the last resolution shell. ^c^Assessed with *MolProbity* (Chen et al., 2010).*

### Other Software

For Cα superpositions and R.M.S.D. calculations the ALIGN program (Cohen, 1997) was used. Figures were prepared in UCSF Chimera (Pettersen et al., 2004).

### Deposition Note

Atomic coordinates and processed structure factors corresponding to the final model of the Hyp-1/melatonin complex have been deposited with the PDB under the accession code 5I8F. The corresponding raw X-ray diffraction images have been deposited in the RepOD Repository at the Interdisciplinary Centre for Mathematical and Computational Modelling (ICM) of the University of Warsaw, Poland, and are available for download with the following Digital Object Identifier (DOI): http://dx.doi.org/10.18150/repod.4711822.

## Results and Discussion

### Crystallization Trials

Hyp-1 co-crystallization experiments were carried out with phytohormones from different classes, including auxin, *trans*-zeatin, gibberellic acid, abscisic acid, and melatonin; and additionally with the flavonoid quercetin, the fluorescence probe ANS, as well as with the hypothetical substrate (emodin) and product (hypericin) molecules. All these trials were performed using the same commercial screens and with similar protein:ligand ratios as for the present complex. The ultimate result of those crystallizations was that crystalline complexes of Hyp-1 could be obtained only with ANS (Sliwiak et al., 2015) or with MEL (added in solution or in powder form). In this context, it is interesting to note that crystallography emerges as a superior approach to the detection of protein-ligand complexes when standard biophysical methods fail (Schiebel et al., 2016).

Competitive crystallization with MEL and *trans*-zeatin resulted in crystals of the same Hyp-1/MEL complex. Thus one can conclude that *trans*-zeatin does not perturb Hyp-1/MEL complex formation under the conditions of Hyp-1/MEL crystal growth.

On the other hand, the presence of ANS in the Hyp-1/MEL crystallization conditions, even at lower concentration than that used for *trans*-zeatin, suppressed the crystal growth entirely. Moreover, addition of melatonin (even at 1:1 ANS:MEL ratio) to Hyp-1/ANS crystallization conditions (Sliwiak et al., 2015) resulted in crystals of the Hyp-1/ANS complex with a new type of modulation (Sliwiak, Unpublished Data). This suggests that ANS blocks the MEL binding sites of Hyp-1 with higher affinity, explaining why it was not possible to detect any signal with MEL titration in ANS Displacement Assays (ADA) performed according to a well-established procedure (Pasternak et al., 2006). On the other hand, it has to be noted that unlike in the Hyp-1/MEL complex, in the crystal structure of the Hyp-1/ANS complex, in addition to the internal binding sites 1,2,3, there are numerous ANS molecules bound at conserved sites on the surface of the Hyp-1 protein (Sliwiak et al., 2015). Since those superficial ANS molecules (which are most likely responsible for the modulation of the crystal structure) are not exchangeable for MEL even at high melatonin concentration, they could additionally mask the ADA signal.

### Overall Features of the Crystal Structure

The structure of Hyp-1 described in this work is of the highest resolution (1.30 Å) among all Hyp-1 structures available in the PDB (3IE5, 1.69 Å; 4N3E, 2.43 Å) and therefore provides the most accurate model of this protein. Moreover, as the protein was purified in reducing condition, in variance with the 3IE5 model, in the present structure there are no accidental disulfide bonds bridging the Hyp-1 molecules in the crystals structure. In agreement with this, PISA (Krissinel and Henrick, 2007) analysis did not detect any stable quaternary structure. The solvent content of the crystal is 56.4% with Matthews volume equal to 2.82 Å^3^/Da. Thanks to the high resolution of the diffraction data, all atoms in the structure were refined with anisotropic atomic displacement parameters (*B*-factors). Careful examination of difference electron density maps revealed the positions of 204 water molecules as well as of two molecules of MEL (**Figure 1B**), one of which (modeled in two orientations, with average *B*-factor of 54.5 Å^2^) is most likely endowed with rotational degrees of freedom within the surface invagination, and another one (modeled in one orientation with <*B*> of 36.1 Å^2^) is well stabilized in the internal cavity of the protein. Two glycerol molecules with low *B*-factors were modeled at the protein surface. In addition, there are three Na^+^ ions included in the model, two of which are octahedrally coordinated by the protein (one by loop L5 and another one by strand β1 and the C-end of helix α3), and a third one partially occupied within the protein cavity. Within the cavity, there is also an ambiguous patch of electron density which could not be assigned to any of the components of the crystallization solution. Since there is an indication of an indole ring with a short side chain (**Figure 1B**), it could be a poorly occupied MEL molecule or a product of its degradation. In view of these doubts, we decided to model this density with several water molecules marked as UNL (Unknown Ligand).

The main chain of the protein model could be traced in electron density without any brakes and it was possible to determine the rotamers of all side chains. Only the last two, one and three atoms, respectively, of three lysine side chains, Lys21, Lys40, and Lys113, which are directed toward bulk solvent, were omitted from the model due to their high mobility and lack of electron density. For 13 residues two rotamers of the side chain could be determined. The structure was refined to *R*/*R*_free_ of 12.8/15.3% and *MolProbity* (Chen et al., 2010) analysis emphasizes the high stereochemical quality of the model (**Table 1**).

### Overall Fold and Three New PR-10 Binding Sites

As already established by Michalska et al. (2010), Hyp-1 has the canonical PR-10-fold with all its structural motifs (**Figure 1A**). The residue ranges of each structural motif are given in **Table 2**. Although the protein main chain creates the typical “baseball glove” framework, the peculiarity of the Hyp-1 protein lies in the side chains, which are responsible for shaping a very interesting and unusual internal cavity, quite different from the cavities known from other PR-10 proteins. As discussed before (Sliwiak et al., 2016), the PR-10 proteins of known structure appear to possess two types of cavities; type I, which is small, shallow, opened at the E1 entrance and capable of binding one ligand molecule in a specific manner; and type II, which resembles a spacious bag and spans the entire hydrophobic core between entrances E1 and E2, and is capable to accommodating more than two ligands of different chemical nature at different positions. In the case of Hyp-1, we observe two separated internal chambers (1 and 2), each with its own entrance (E1 and E2, respectively), and a third site (3) which is formed as a deep invagination of the protein surface. A very unusual feature of the Hyp-1 binding sites as compared to other PR-10 proteins is the amazing conservation of the ligand position; regardless of their chemical character, the ligand molecules always take the same position in the three sites, as clearly illustrated by the cut-away sections of the protein interior in **Figure 1C**.

**Table 2:** Residue ranges of PR-10 canonical motifs in Hyp-1 protein.

Secondary structure element	No	Residue range
α-helix	1	Pro16–Leu23
	2	Arg27–Ala34
	3	Glu130–Asn154
β-sheet	1	Ala2–Ser12
	2	Ser41–Glu46
	3	Val54–Thr58
	4	Tyr67–Asp76
	5	Tyr81–Glu88
	6	Lys98–Leu105
	7	Lys113–His121
Loop	1	Pro13–Ala15
	2	Val24–Glu26
	3	Gln35–Lys40
	4	Gly47–Thr53
	5	Phe59–Thr66
	6	Ala77–Phe80
	7	Gly89–Glu97
	8	Glu106–Ser112
	9	Pro122–Asn129
Unstructured	–	Pro155–Ala159

**FIGURE 3:**
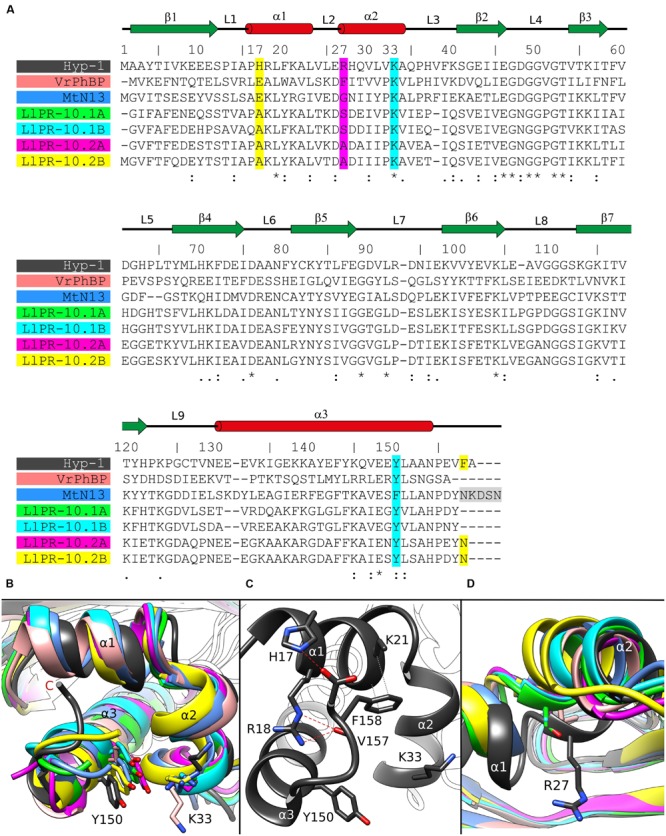
**(A)** Multiple sequence alignment and **(B)** superposition of PR-10 models (identified by their PDB codes and color) with zoom on the area of the Lys33–Tyr150 invagination of Hyp-1. Green, LlPR-10.1A (4RYV); cyan, LlPR-10.1B (1IFV, chain A); magenta, LlPR-10.2A (1XDF, chain B); yellow, LlPR-10.2B (2QIM); salmon, VrPhBP (2FLH, chain B); blue, MtN13 (4JHG); dark gray, the present model. In the sequence alignment **(A)**, the positions corresponding to Hyp-1 Pro17, Arg27, Lys33, Tyr150 and Phe158 are highlighted as follows: cyan, conservative residues creating the surface invagination; yellow, unique Hyp-1 residues that are involved in C-end stabilization; magenta, the cavity separator Arg27. The disordered C-terminal pentapeptide of MtN13 is highlighted in gray. Identical (^∗^) as well as more (:) and less (.) similar residues are marked at the bottom, while the pictograms above the Hyp-1 sequence numbers illustrate the secondary structure elements (green arrows, β-strands; red cylinders, α-helices) and their annotation. **(C)** Interaction of the C-end of Hyp-1 with α1, stabilizing the novel binding site 3. **(D)** Cα superposition of different PR-10 models as in **(B)** with zoom on the α2 structural element, with residues corresponding to Hyp-1 Arg27 shown in stick representation.

The binding site 3 is quite mysterious. Although the residues stabilizing the ligand in a vise-type manner (Lys33 and Tyr150) are conserved among almost all PR-10 proteins, Hyp-1 is the only protein where ligand binding is found at this site and is seen there in all available structures of Hyp-1 complexes. An explanation of this observation may lie in the interaction between the unstructured C-terminal end of Hyp-1 and helix α1. Among the aligned sequences (**Figure 3A**) of PR-10 proteins studied structurally in our laboratory, Hyp-1 is the only one to have a long C-terminal peptide forming a C-terminal loop that interacts with helix α1 (**Figure 3B**). This interaction involves hydrogen bonds between the N𝜀2 atom of His17 and the C-terminal carboxylate group (of Ala159) and between the N𝜀 and Nη2 atoms of Arg18 and the O atom of Val157, as well as hydrophobic interactions of the aromatic ring of Phe158 with Lys21 and the main chain of helix α1 (**Figure 3C**). His17 is unique to the Hyp-1 protein, as in other PR-10 proteins there is a negatively charged Glu or hydrophobic Ala residue at this position (**Figure 3A**). Although the C-terminal sequence of MtN13 is even longer than in Hyp-1 (**Figure 3A**), in all crystal structures of MtN13/cytokinin complexes (4GY9, 4JHG, 4JHH, 4JHI; Ruszkowski et al., 2013), this end of the protein is disordered, indicating the absence of such C-terminal stabilization. The interactions mentioned above stabilize the surface invagination of Hyp-1, thereby creating the new ligand binding site 3. The above interactions between the C-terminus and helix α1 are present in all experimental models of Hyp-1.

As discussed before (Sliwiak et al., 2015), the main partition between the chambers 1 and 2 in Hyp-1 is the long side chain of Arg27 from helix α2, with further contribution from Tyr84, Tyr101, Ala140, and Phe143. A structural superposition of the α2 helix of different PR-10 proteins (**Figure 3D**) reveals not only that the Hyp-1-specific Arg27 residue is replaced in other PR-10 sequences by Gly, Ala or Ser, but also that the α2 helix of Hyp-1 penetrates the hydrophobic core in an exceptionally high degree, contributing to this unique partitioning into two separate internal chambers.

### Ligand Identification in Electron Density

As mentioned above, MEL1 is the best stabilized ligand in the structure. Its electron density (**Figure 1B**) clearly indicates the position of each atom. A similar situation was found in the Hyp-1/ANS complex, where the superposition of the 28 Hyp-1 molecules in the asymmetric unit produced an exceptionally consistent overlay of the ligand molecules at site 1 (Sliwiak et al., 2015). We note, however, that there is a strange positive electron density peak less than 2 Å from the MEL1 methoxy group (**Figure 1B**), for which we do not have a plausible explanation.

Although we did not model melatonin at site 2, it is quite obvious that the electron density there is consistent with the shape of the indole ring. However, we were unable to build a satisfactory model of MEL or 5-methoxyindole there. One possibility is that the ligand at site 2 is very mobile. Alternatively, a melatonin degradation product could be bound there. As a free radical scavenger, melatonin is rather unstable and could undergo X-ray-induced degradation. To test this possibility, we irradiated an NMR probe containing 0.6 M solution of melatonin in deuterated methanol with a synchrotron X-ray dose ∼10 times higher than that used in the diffraction experiment. However, the NMR spectrum after irradiation was unchanged, suggesting that photodegradation was not a likely mechanism of the observed effect. Notwithstanding this result, we were also unable to model N1-acetyl-N2-formyl-5-methoxykynuramine (AFMK) or 6-hydroxymelatonin at this site, the two known photodegradation products of melatonin (Maharaj et al., 2002).

MEL3 has flat electron density, indicating in-plane rotation of the ligand. To account for this effect, we modeled MEL3 in two orientations. It is interesting to note that rotation of the flat ANS molecule at site 3 site was also observed in the Hyp-1/ANS complex, with the caveat that the rotation could be deduced from the superposition of the 28 copies of the Hyp-1 molecule, whereas in each individual case the ANS3 ligand could be modeled in a unique orientation. Nevertheless, despite the rotation of MEL3, it is safe to conclude that the ligand molecule is firmly docked between the jaws of the Lys33–Tyr150 vise.

It is very important to stress that the ligand electron densities described above were perfectly reproducible in several independent structure determinations utilizing differently produced Hyp-1/MEL crystals, namely either in the presence of solid (pulverized) melatonin or in the presence of equimolar concentration of *trans*-zeatin. The reproducibility includes even the inexplicable electron density peak near the MEL1 molecule.

### Ligand Binding

The MEL molecule at site 1 that has the best definition in electron density, makes direct contacts with the protein only via weak (3.6–3.8 Å) hydrophobic interactions with Phe39, Leu31, Leu65, Val91, Gly136, and Lys139, as well as via water-mediated hydrogen bonds of its O2^1^ atom with Gly136 (O), Ala140 (N), and Met68 (Sδ), and of its N1 atom with Arg93 (Nη2) and Glu132 (O).

**FIGURE 4:**
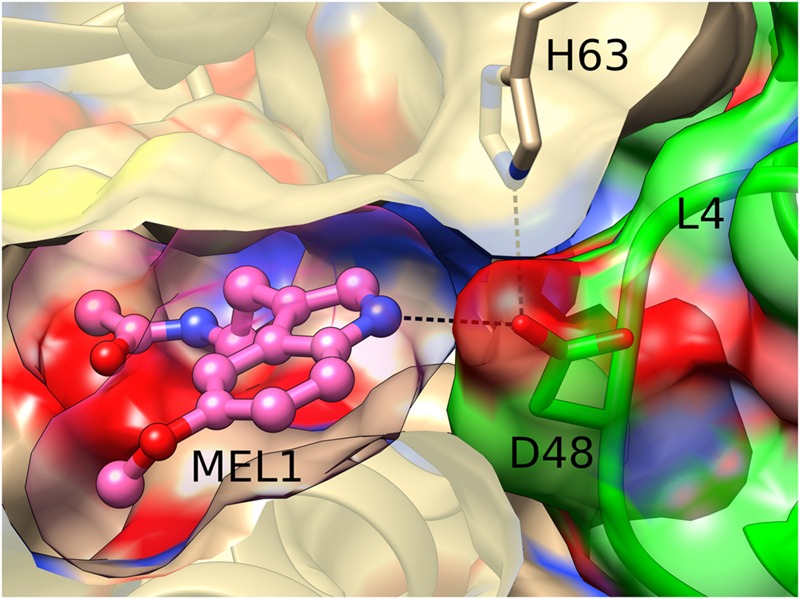
**Hydrogen bonding between MEL1 and Asp48 from loop L4 of an adjacent Hyp-1 molecule (green).** MEL1 is shown as a ball-and-stick model surrounded by its (semitransparent) van der Waals surface, and the two interacting Hyp-1 molecules (sand and green) are presented with their semitransparent van der Waals surfaces.

Interestingly, the MEL1 ligand is additionally stabilized and pushed to its binding site by a direct hydrogen bond of its N2 atom with the carboxylate group of Asp48 from loop L4 of an adjacent Hyp-1 molecule. This interaction is additionally stabilized by a hydrogen bond between the “intruding” Asp48 carboxylate and the N𝜀 atom of His63 from loop L5 of the MEL1-binding protein molecule (**Figure 4**). Such a situation is not new among PR-10 proteins. In the structures of *Medicago truncatula* Nodulin 13 (MtN13) in complex with cytokinins (Ruszkowski et al., 2013), there is a similar interaction with Asp62 from loop L5 of another copy of MtN13, which forms a fork of hydrogen bonds with the N6 and N7 atoms of the cytokinin molecule. In variance with the Hyp-1 situation, however, the cytokinin...Asp62 interaction in MtN13 is mutual, leading to (quite exceptional) dimer formation of that PR-10 protein.

As mentioned above, the MEL3 ligand is evidently rotating between the jaws of the vise formed by the side chains of Lys33 and Tyr150. Its stacking interactions with these residues have van der Waals character.

The unidentified ligand marked by dummy (UNL) water molecules at site 2 is in van der Waals distance to residues Leu19, Ile116, and Tyr144. The same residues stabilize the ANS2 ligand in the binding site 2 of the Hyp-1/ANS complex (Sliwiak et al., 2015).

### Conformational Differences between the Available Hyp-1 Complexes

**FIGURE 5:**
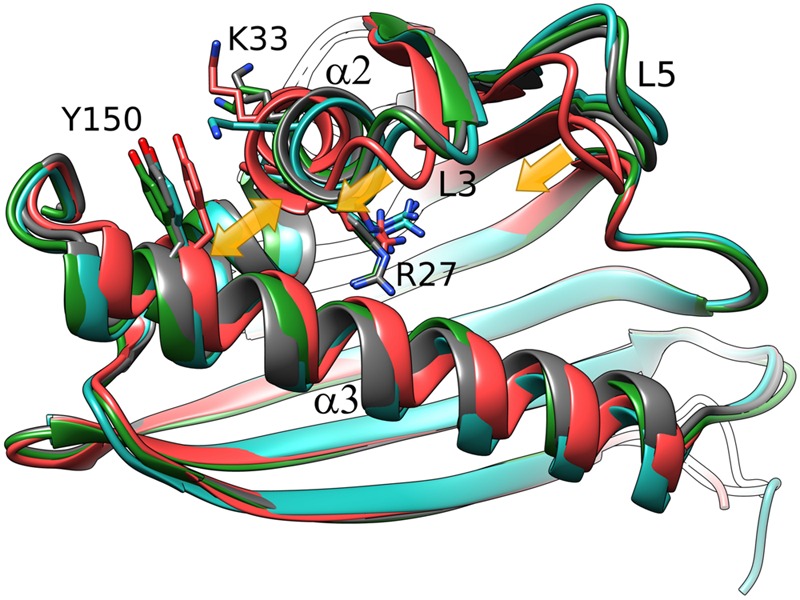
**Cα superposition of the available models of Hyp-1.** Color code: Hyp-1/MEL (this work), dark gray; Hyp-1/ANS (4N3E, chain K), green; Hyp-1/PEG (3IE5), chain A – red, chain B – blue. Yellow arrows indicate the conformational rearrangements in chain A of 3IE5, which has an empty site 3, in particular the approach of helices α2 and α3 (highlighted by Tyr150) leading to a tighter grip of the fingertip loops L3 and L5.

We note at the outset that there is no truly ligand-free structure of Hyp-1 in the PDB. The closest case of a protein crystallized without any intentional ligand is 3IE5 (Michalska et al., 2010) but even in that structure there are serendipitous PEG molecules found in the binding sites of the two protein chains A and B. A superposition of the Cα atoms revealed that the structures of Hyp-1 complexed with melatonin, ANS (4N3E, represented by chain K) and PEG (3IE5, chains A and B) are quite similar, with R.M.S.D. values within ∼1 Å (**Table 3**). That means that the chemical character of the ligand does not influence the Hyp-1-fold to a significant degree and that the three conserved binding sites are capable of accommodating different hydrophobic and amphiphilic ligands from the aqueous environment. Interestingly, chain A of the 3IE5 structure seems to differ most significantly from all the remaining Hyp-1 models, even when compared with chain B from the same structure (**Figure 5**). This difference can be correlated with the fact that in chain A of 3IE5 the binding site 3 is empty, allowing Tyr150 and Lys33 to form a direct stacking contact. This interaction brings the end of helix α3 closer to helix α2 and, in consequence, pulls the loops L3 and L5 toward α3. One can speculate that binding of a ligand molecule at site 3 of Hyp-1 widens the E1 entrance, facilitating access of another ligand molecule to site 1. We can therefore hypothesize that the ligand binding mechanism of Hyp-1 has a cooperative character. Moreover the PEG molecules in 3IE5 (**Figure 5**) seem to pull the main cavity separator (Arg27) away from the hydrophobic core, resulting in a less solid separation between chambers 1 and 2.

**Table 3:** R.M.S.D. (Å) values of Cα superpositions of the following Hyp-1 models: Hyp-1/MEL complex (this work), Hyp-1/ANS complex (4N3E, chain K), and chains A/B from the “ligand free” (i.e., Hyp-1/PEG) form (3IE5).

	3IE5, chain A	3IE5, chain B	4N3E, chain K
Hyp-1/MEL	1.01	0.72	0.61
4N3E, chain K	1.04	0.75	
3IE5, chain B	1.07	

## Conclusion

Hyp-1, a protein from *H. perforatum*, has the characteristic PR-10-fold. However, despite the overall similarity, it has three highly unique and characteristic ligand binding sites, which may suggest a unique ligand-binding mechanism among the PR-10 proteins. Although the interaction of Hyp-1 with melatonin does not appear to be particularly strong, the structure of the Hyp-1/MEL complex is quite robust and reproducible in a number of crystal structure determination experiments. The reproducibility regards also the unidentified electron density at site 2 and an unattributed peak near the methoxy group of MEL1. Moreover, co-crystallization trials with other phytohormones and natural ligands using the same crystallization screens as in the Hyp-1/MEL experiments, produced no results. The three binding sites identified in the Hyp-1/MEL complex are exactly the same as in the Hyp-1/ANS complex. They comprise a well ordered MEL (site 1), a rotationally disordered one (3) and possibly an unidentified melatonin degradation product (2). Considering all the facts together, one can conclude that Hyp-1 may be capable of melatonin storage/transport under stress conditions in *H. perforatum*. A shortlist of the supporting observations is as follows: (i) Hyp-1, as a probable pathogenesis-related protein (a superfamily, whose members are expressed *inter alia* during abiotic and biotic stress) was detected in the roots and other parts of *Hypericum* plantlets and its mRNA expression has the highest level in the roots. (ii) Melatonin concentration is very high in vulnerable parts, like seedlings, as well as in the leaves and flowers of mature *Hypericum* plants, and it is further elevated during, e.g., radiation stress, and thus it could be bound by Hyp-1 despite of a relatively low affinity. (iii) Melatonin and its precursor tryptophan have been reported to be absorbed by plant roots from soil and media (Tan et al., 2007). (iv) *trans*-Zeatin, which is frequently reported as a natural ligand of PR-10 proteins, did not affected Hyp-1/MEL crystallization, while the artificial fluorescent probe ANS - did. However, taking into account that some PR-10 proteins show pleiotropic binding capacity (Sliwiak et al., 2016), it should not be ruled out that Hyp-1 may also play other role(s) in *H. perforatum*.

## Author Contributions

JS designed the experiments, conducted the experiments and calculations, analyzed the results, drafted the manuscript. ZD supervised initail experiments, conducted X-ray diffraction experiments and data processing, participated in ms preparation. MJ supervised the project, analyzed the results, wrote the paper.

## Conflict of Interest Statement

The authors declare that the research was conducted in the absence of any commercial or financial relationships that could be construed as a potential conflict of interest.

## Footnotes

1 The atom numbering scheme of melatonin (**Figure 2A**) follows that of Quarles et al. (1974) because the system used by the PDB is quite irrational and inconsistent with established rules, as also noted for other ligands (Jaskolski, 2013).
